# *Strongyloides stercoralis* is associated with significant morbidity in rural Cambodia, including stunting in children

**DOI:** 10.1371/journal.pntd.0005685

**Published:** 2017-10-23

**Authors:** Armelle Forrer, Virak Khieu, Fabian Schär, Jan Hattendorf, Hanspeter Marti, Andreas Neumayr, Meng Chuor Char, Christoph Hatz, Sinuon Muth, Peter Odermatt

**Affiliations:** 1 Department of Epidemiology and Public Health, Swiss Tropical and Public Health Institute, Basel, Switzerland; 2 University of Basel, Basel, Switzerland; 3 National Centre for Parasitology, Entomology and Malaria Control, Ministry of Health, Phnom Penh, Cambodia; 4 Department of Medicine, Swiss Tropical and Public Health Institute, Basel, Switzerland; Centre for Tropical Diseases, ITALY

## Abstract

**Background:**

*Strongyloides stercoralis* is a soil-transmitted nematode that can replicate within its host, leading to long-lasting and potentially fatal infections. It is ubiquitous and highly prevalent in Cambodia. The extent of morbidity associated with *S*. *stercoralis* infection is difficult to assess due to the broad spectrum of symptoms and, thus, remains uncertain.

**Methodology/Principal findings:**

Clinical signs were compared among *S*. *stercoralis* infected *vs*. non-infected participants in a cross-sectional survey conducted in 2012 in eight villages of Northern Cambodia, and before and after treatment with a single oral dose of ivermectin (200μg/kg BW) among participants harboring *S*. *stercoralis*. Growth retardation among schoolchildren and adolescents was assessed using height-for-age and thinness using body mass index-for-age. *S*. *stercoralis* prevalence was 31.1% among 2,744 participants. Urticaria (55% *vs*. 47%, OR: 1.4, 95% CI: 1.1–1.6) and itching (52% *vs*. 48%, OR: 1.2, 95% CI: 1.0–1.4) were more frequently reported by infected participants. Gastrointestinal, dermatological, and respiratory symptoms were less prevalent in 103 mono-infected participants after treatment. Urticaria (66% *vs*. 11%, OR: 0.03, 95% CI: 0.01–0.1) and abdominal pain (81 *vs*. 27%, OR: 0.07, 95% CI: 0.02–0.2) mostly resolved by treatment. *S*. *stercoralis* infection was associated with stunting, with 2.5-fold higher odds in case of heavy infection.

**Conclusions/Significance:**

The morbidity associated with *S*. *stercoralis* confirmed the importance of gastrointestinal and dermatological symptoms unrelated to parasite load, and long-term chronic effects when associated with malnutrition. The combination of high prevalence and morbidity calls for the integration of *S*. *stercoralis* into ongoing STH control measures in Cambodia.

## Author summary

*Strongyloides stercoralis* is an intestinal parasite that infects humans by penetrating intact skin. It thrives particularly in tropical countries with poor sanitation. Because it can replicate within its host, it causes long-lasting infections and is potentially fatal in patients with a disseminated infection. *S*. *stercoralis* is largely neglected due to the difficulty in detecting it with standard field diagnostic techniques but has recently been found to be very common in Cambodia, with prevalence rates exceeding 40%. It is difficult to identify symptoms associated with infection in endemic areas because co-infections with other helminths or protozoan parasites, which cause similar health problems, are common. We compared clinical signs in infected *vs*. non-infected participants living in eight villages in Northern Cambodia, and before and after treatment with ivermectin, the drug of choice against *S*. *stercoralis*, among 103 patients infected with *S*. *stercoralis* only. We also assessed the association between infection and growth retardation among children and adolescents. Of the participants, 31.1% were infected with *S*. *stercoralis*. Infected participants were more likely to report itching and urticaria. After treatment, fewer participants reported urticaria, abdominal pain, vomiting and, to a lesser extent, nausea, diarrhea, cough, and tiredness. *S*. *stercoralis* infection was associated with growth retardation as expressed by stunting.

## Introduction

*Strongyloides stercoralis*, one of the most difficult to diagnose neglected tropical diseases, is an intestinal soil-transmitted parasitic nematode that occurs worldwide and is highly prevalent in warm regions with poor sanitation [1, 2]. Its prevalence is largely underestimated due to the inability of simple coprological diagnostic techniques to detect *S*. *stercoralis* larvae [3, 4]. Although numerous aspects of the epidemiology of *S*. *stercoralis* remain poorly documented, the parasite is very common, with prevalence rates in the tropics and subtropics exceeding 40% [1]. A rough estimate of 200–370 million cases worldwide has recently been put forward [3].

Because it can be life-threatening in immunocompromised patients, *S*. *stercoralis* is also known throughout the developed countries, with most literature originating from hospital-based case reports of severe strongyloidiasis among transplant recipients, travelers, and migrants. Commonly reported symptoms of uncomplicated strongyloidiasis include diarrhea, vomiting, abdominal pain, urticaria, and “larva currens”, while half of the cases are asymptomatic [5–7]. Larva currens is an intermittent urticarial linear, serpiginous eruption due to the migration of larvae under the skin. The high speed at which larvae travel (5 to 10 centimetres per hour) and the location of lesions (lower trunk, bottom and thighs) make larva currens a highly specific symptom of *S*. *stercoralis* infection [5, 8, 9].

*S*. *stercoralis* can replicate within its host, permitting ongoing “autoinfection”. This leads to long-lasting infections and potential fatalities among immunosuppressed patients, such as those undergoing corticosteroid therapy or suffering certain concomitant diseases or malnutrition [2, 5, 10]. There is a paucity of studies investigating the health impacts of *S*. *stercoralis* in low-income tropical and sub-tropical countries, including its associations with malnutrition and growth retardation [2, 4]. It is challenging to document the clinical signs and symptoms associated with specific soil-transmitted helminth (STH) infections in endemic poly-parasitic settings because of their non-specific clinical presentations and because of the difficulty accounting for co-infections (due to the sub-optimal sensitivity of most direct diagnostic approaches) [4].

Preventive chemotherapy, the strategy for controlling STH, would be feasible for *S*. *stercoralis* using a single oral dose of ivermectin (200 μg / kg body weight (BW)), which achieves high cure rates, comparable to that of a two dose regimen [11–16].

The evidence base for morbidity in endemic areas is small and must be improved in order to have *S*. *stercoralis* integrated into the WHO control strategy for reducing the global burden of soil-transmitted helminths [2–4]. The objective of this work was to quantify the morbidity associated with chronic *S*. *stercoralis* infection in a highly endemic setting in Cambodia. We compared infected *vs*. non-infected participants and assessed the degree of symptom resolution achieved with a single oral dose of ivermectin (200 μg / kg BW), while excluding or adjusting for co-infection with other helminth species or pathological protozoan parasites. Additionally, we investigated the association between growth retardation and *S*. *stercoralis* infection risk and intensity among schoolchildren and adolescents.

## Methods

### Ethics statement

The study protocol was approved by the National Ethics Committee for Health Research, Ministry of Health, Cambodia; and by the Ethics Committee of Northeast and Central Switzerland. All participants were informed of the study purpose and procedures and written informed consent was obtained before enrollment.

### Study area, design and population

Morbidity associated with *S*. *stercoralis* infection was assessed in three sub-studies. First, symptoms associated with *S*. *stercoralis* infection were identified in a community-based cross-sectional study. Second, symptom resolution by standard treatment was quantified in a before-and-after treatment approach. Third, the association between malnutrition and *S*. *stercoralis* infection was assessed for children participating in the cross-sectional study.

A community-based, cross-sectional survey was conducted between February and June 2012 in Rovieng district, Preah Vihear Province, Northern Cambodia, where *S*. *stercoralis* is highly endemic [17]. The survey was part of a larger two-year intervention study, described in detail elsewhere [13]. In brief, we included all households in the eight villages that had never received ivermectin treatment; all household members over the age of two were eligible. The resulting sample was used to assess the symptoms associated with *S*. *stercoralis* infection while adjusting for co-infection with any other diagnosed helminths or pathological protozoan parasites. All *S*. *stercoralis* cases were treated with a single oral dose of ivermectin (200μg/kg BW) and all other diagnosed parasitic infections were treated in accordance with the national guidelines [18].

Symptom resolution after ivermectin treatment was investigated among *S*. *stercoralis* infected patients from two villages who were followed-up 21 days after treatment with a single oral dose of ivermectin (200μg/kg BW). Assessment was conducted both among patients with *S*. *stercoralis* mono-infection (i.e. excluding co-infection with any other diagnosed helminths or pathological protozoan parasites) and among all *S*. *stercoralis* infected patients (i.e. all *S*. *stercoralis* cases whether they were co-infected with other parasites or not). Adjustments were made for those with co-infections. A time span of 21 days is long enough to maximize cure time and short enough to avoid reinfection. The single oral dose was chosen because it achieves a high cure rate and is appropriate in the framework of control efforts [13]. Post-treatment data were collected through parasitological assessment and medical interviews about symptoms experienced in the three days preceding follow-up.

The growth retardation assessment was performed among children 5–19 years of age, residing in the eight villages.

### Clinical assessment

Demographic data (sex, age, occupation and education level) and history of anthelmintic treatment were collected from all participants using a pre-tested questionnaire. A clinical assessment of all participants was conducted by a medical doctor and included anthropometric measures, physical examination, and an assessment of signs and symptoms experienced in the two-weeks prior. Information on ownership of household-assets was obtained from heads of households.

### Assessment of parasitological infection

*S*. *stercoralis* was diagnosed using Koga Agar plate (KAP) culture and the Baermann technique on two samples collected on consecutive days [17, 19, 20]. KAP and Baermann centrifuged eluents were checked for species to prevent the misclassification of hookworm *vs*. *S*. *stercoralis* larvae. Other STH were diagnosed using a Kato-Katz thick smear on each of the two fecal samples and formalin-ether concentration technique (FECT) on one sample [21, 22]. Protozoa were diagnosed with FECT performed on one stool sample [22].

*S*. *stercoralis* parasite load was estimated based on larvae counts from the Baermann test, using the following thresholds: low parasite load—a positive count, up to one larva per gram of stool (LPG); moderate parasite load—two to nine LPG; and high parasite load—more than 10 LPG [23, 24]. Participants with positive KAP and negative Baermann tests were assumed to have low parasite loads.

To keep all the positives in the sample and to maximize the specificity, the infection status of participants in any of the three assessments was determined using the following case definition: a *S*. *stercoralis* patient had at least one *S*. *stercoralis* positive fecal sample. A *S*. *stercoralis*-free participant had negative results in all four available diagnostic results (one Baermann and one KAP on each of two samples). The same approach was used for the other helminths, i.e. all positives (determined by at least one positive diagnosis result by any method) and only negatives with two Kato-Katz results were included. Since there was only one FECT result per participant, the case definition does not apply to protozoa.

### Data management and statistical analysis

Clinical and laboratory data were managed and analyzed in STATA version 13.0 (StataCorp LP; College Station, United States of America). Anthropometric measures and summaries were calculated using the WHO code (http://www.who.int/growthref/tools/en/) in R [25]. In the cross-sectional study, age of participants was categorized into four groups, as follows: (i) < 6 years, (ii) 6–18 years, (iii) 19–59 years, and (iv) ≥ 60 years. The age of children included in the growth retardation analysis was categorized into three groups, corresponding to school level (i.e. primary, secondary, high school), as follows: (i) 5–9 years, (ii) 10–13 years and (iii) 14–19 years.

First, the association between each symptom, *S*. *stercoralis* infection status, and parasite load was investigated using multivariate logistic regression models (with the symptom as outcome), adjusting for any other helminthic or protozoan infection, sex, age, and treatment uptake in the past year.

Second, the symptom resolution achieved by ivermectin treatment was assessed with McNemar’s exact test among patients harboring *S*. *stercoralis* mono-infections, by comparing the proportions of participants with a particular symptom before and after treatment. An additional analysis including co-infections with other parasites used conditional logistic regression models with each symptom as outcome, and survey (before *vs*. after treatment) and presence of any other infection as explanatory variables.

Third, for assessing growth retardation and thinness in children, the z-scores for height-for-age (HAZ) and body mass index (BMI)-for-age (BAZ) were calculated for children between 5 and 19 years, participating in the cross-sectional survey. The WHO Growth Reference Standard was used to calculate anthropometric indicators for school-aged children and adolescents [26, 27].

Children with HAZ and BAZ values lower than -2 were classified as “stunted” or “thin”, respectively, as opposed to “normal” for values larger than -2. The association between stunting, thinness and *S*. *stercoralis* infection status and parasite load was assessed using logistic regression with the nutritional variable as outcome. The final multivariate model was built based on Akaike Information Criterion (AIC). Interactions between sex and *S*. *stercoralis* parasite load, age, and socioeconomic status, as well as between age and *S*. *stercoralis* parasite load and socioeconomic status were checked using the Likelihood Ratio Test (LRT).

## Results

### Study population

Among the 3,837 participants enrolled in the study, diagnostics could not be performed for *S*. *stercoralis* or for protozoan infections for 134 and 320 individuals, respectively, due to absent samples (participants did not return any sample) or an insufficient amount of stool to perform the FECT. The remaining 3,377 participants all completed the interview and the clinical assessment and were included in the study. Females and children under six years old were significantly more prone to missing diagnostics and exclusion from the sample. All regression models were adjusted for sex and age, which controlled for this potential bias.

**Fig 1:**
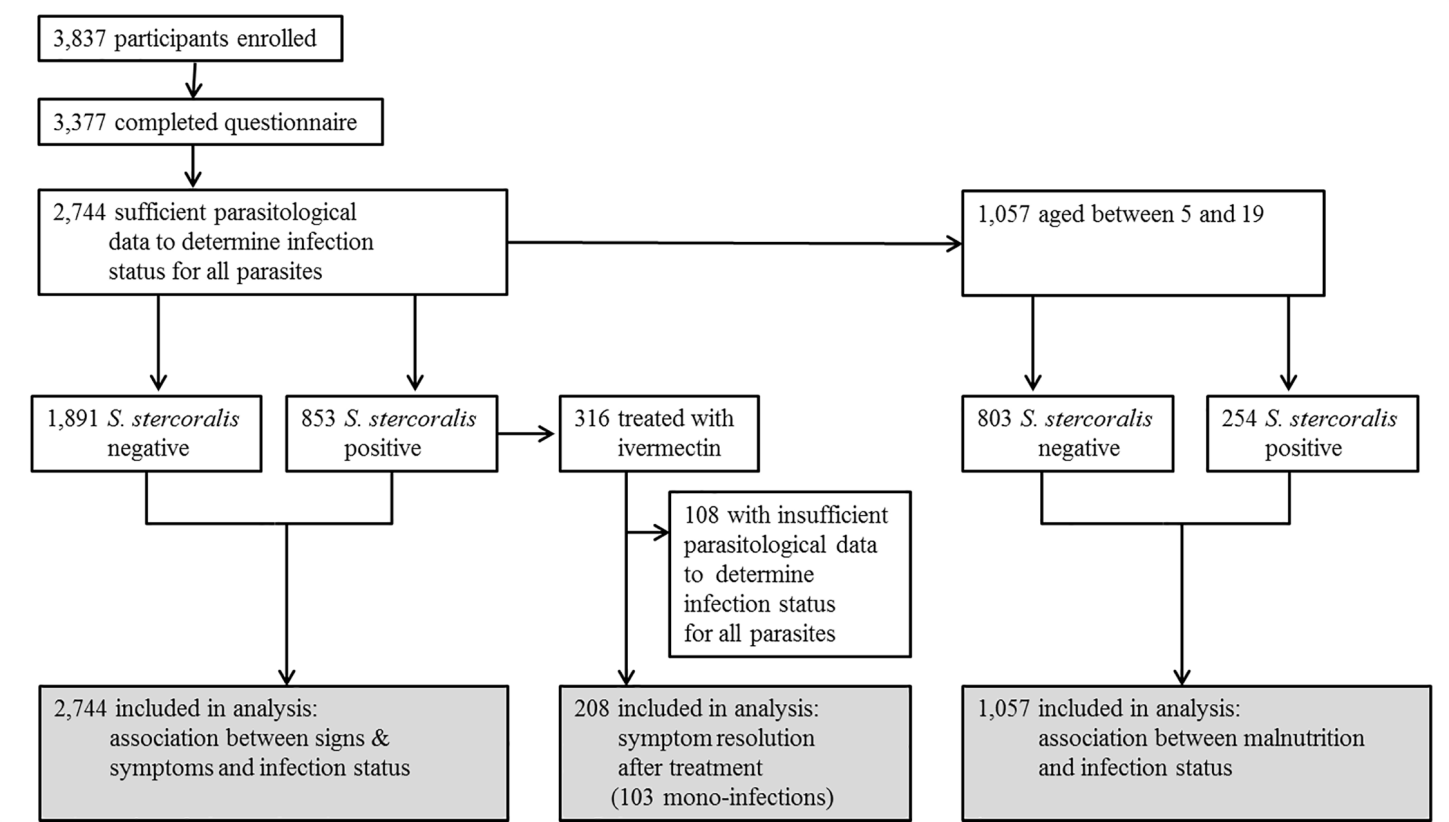
Study flow chart. Flowchart detailing the number of participants included in the three sub-studies. Insufficient parasitological data corresponds to one (out of four) or more missing diagnostic examinations for *S*. *stercoralis*, to one (out of two) or more missing diagnostic examinations for other helminths, and to any missing diagnostic examination for protozoa.

The association between *S*. *stercoralis* infection and symptoms was assessed for 2,744 participants with confirmed infection status for all investigated parasites (Fig 1). This final sample size results from excluding all participants with uncertain negative status for helminth infection, i.e. those with fewer than four negative results (two methods applied to two samples) for *S*. *stercoralis* and fewer than two negative results (Kato Katz on two samples) for other helminths, to maximize diagnosis specificity. Basic characteristics of this sample are presented in Table 1. The prevalence of *S*. *stercoralis* infection was 31.1% (95%CI: 29.4–32.9). A quarter (699/2,744; 25.3%) of participants was infected with hookworm, which was the only other common parasite. Among *S*. *stercoralis* infected patients, 325/853 (38.1%) were co-infected with hookworm. Other helminths species were rare, with prevalence rates below 3%, while 8% of participants were infected with pathogenic protozoa, namely *Giardia lamblia* or *Entamoeba histolytica/dispar*. Species specific prevalences are available in S1 Table.

**Table 1:** Characteristics of participants included in the three analyzed samples.

Variable	Category	n (%)
**Sample "positives *vs*. negatives"**		**N = 2,744**
Sex	Male	1,240 (45.2)
	Female	1,504 (54.8)
Age (years)	< 6	203 (7.4)
	6–18	982 (35.8)
	19–59	1,379 (50.3)
	≥ 60	180 (6.5)
Anthelmintic treatment in the past year	No or don’t know	1,015 (37.0)
	Yes	1,729 (63.0)
Occupation	Rice farmer	1,397 (50.9)
	At home	296 (10.8)
	School	945 (34.4)
	Tertiary, business, other	106 (3.9)
**Sample "before-after"**		**N = 103**
Sex	Male	55 (53.4)
	Female	48 (46.6)
Age (years)	< 6	3 (2.9)
	6–18	22 (21.4)
	19–59	68 (66.0)
	≥ 60	10 (9.7)
Anthelmintic treatment in the past year	No or don’t know	50 (48.5)
	Yes	52 (51.5)
Occupation	Rice farmer	64 (62.1)
	At home	7 (6.8)
	School	21 (20.4)
	Tertiary, business, other	11 (10.7)
**Sample "childhood malnutrition"**		**N = 1,057**
Sex	Male	535 (50.6)
	Female	522 (49.4)
Age (years)	5–9	339 (32.1)
	10–13	364 (34.4)
	14–19	354 (33.5)
Anthelmintic treatment in the past year	No or don’t know	394 (37.3)
	Yes	663 (62.7)
Occupation	School	904 (85.5)
	At home, other	90 (8.5)
	Rice farmer	63 (6.0)

Sample "positives *vs*. negatives": cross-sectional sample for assessing the association between *S*. *stercoralis* infection and clinical signs.

Sample "before-after": sample for assessing clinical signs present before and after a single oral dose of ivermectin (200 μg/kg BW) in patients infected with *S*. *stercoralis* only.

Sample "children malnutrition": sample for assessing the association between stunting and *S*. *stercoralis* infection.

For the before-after treatment assessment, 208 of the 316 *S*. *stercoralis* positive participants had confirmed infection status for all parasites at both assessments. About half (103/208) of the participants in that sub-sample were not infected with any other helminth or pathogenic protozoa either before or after treatment, while the other 105 harbored other intestinal parasites before and/or after treatment. Basic characteristics of the sample analyzed for the before-after assessment are presented in Table 1.

The growth retardation assessment was conducted among 1,057 children aged 5–19 years, with confirmed infection status for *S*. *stercoralis* and hookworm, and who had *S*. *stercoralis* parasite load assessed. Of the 1,338 children aged 6–19 years participating in the study, 229 were excluded from the final analysis due to incomplete diagnostics for *S*. *stercoralis*, seven were excluded because of missing diagnosis results for hookworm, and 47 were excluded due to the absence of parasite load information. Basic characteristics of the sample are presented in Table 1. Among this group, the prevalence of *S*. *stercoralis* was 24.0% (95%CI: 21.5–26.7). Most cases (160/254; 63.0%) had low parasite loads, whereas 20.1% and 16.9% had moderate and heavy parasite loads, respectively.

### Symptoms associated with *S*. *stercoralis* infection

Overall, only five (1.1%) participants with *S*. *stercoralis* mono-infection and 18 (1.4%) infection-free participants were reportedly without symptoms. The most frequently reported symptoms by *S*. *stercoralis*-infected patients were abdominal pain, cough, epigastric pain, diarrhea, and urticaria, ranging from 83.1% to 55.3%. However, in most cases they were not significantly associated with *S*. *stercoralis* infection. The symptoms with the strongest association were urticaria (55.3% *vs*. 46.5%, OR: 1.35, 95% CI: 1.13–1.60) and itching (52.4% *vs*. 47.7%, OR: 1.19, 95% CI: 1.00–1.41). The frequency of symptoms and the strength of their association with *S*. *stercoralis* infection status are presented in Table 2.

**Table 2:** Association between symptoms and *S*. *stercoralis* infection among 853 positive and 1,891 negative participants.

			Association with *S*. *stercoralis* infection		
Symptom	*S*. *stercoralis* positive n (%)	*S*. *stercoralis* negative n (%)	OR^(a)^	95% CI	LRT p-value
Loss of appetite / Anorexia	277 (32.5)	598 (31.6)	0.97	0.80–1.17	0.73
Nausea	291 (34.1)	611 (32.3)	1.02	0.85–1.22	0.86
Vomiting	181 (21.2)	380 (20.1)	1.05	0.85–1.29	0.67
Abdominal pain	709 (83.1)	1,479 (78.2)	1.11	0.88–1.41	0.36
Epigastric pain	531 (62.3)	1,085 (57.4)	0.98	0.81–1.20	0.85
Diarrhea	513 (60.1)	1,086 (57.4)	1.10	0.93–1.31	0.28
Constipation	128 (15.0)	293 (15.5)	0.91	0.71–1.15	0.42
Cough	533 (62.5)	1,208 (63.9)	1.00	0.84–1.19	0.98
Wheezing	72 (8.4)	147 (7.8)	1.03	0.76–1.41	0.85
Itching	447 (52.4)	902 (47.7)	**1.19**	1.00–1.41	0.05
Urticaria	472 (55.3)	880 (46.5)	**1.35**	1.13–1.60	0.001
Generalized rash	186 (21.8)	345 (18.2)	1.20	0.97–1.48	0.09
Fever	360 (42.2)	855 (45.2)	0.98	0.83–1.17	0.85
Tiredness	205 (24.0)	421 (22.3)	1.04	0.84–1.28	0.75
Muscle pain	381 (44.7)	688 (36.4)	1.17	0.97–1.40	0.10

^(a)^ Odds ratios were adjusted for age, sex, medication (uptake of anthelmintic tablets within the past year), and infection with any diagnosed helminth or pathogenic protozoa.

OR in bold were significant at 5% level.

OR: odds ratio.

CI: confidence interval.

LRT: likelihood ratio test

When accounting for *S*. *stercoralis* parasite load instead of infection status, urticaria was also found to be significantly associated, but not itching (S2 Table). Of note, age was associated with most of the reported symptoms (S3 Table).

### Symptom resolution after treatment

**Fig 2:**
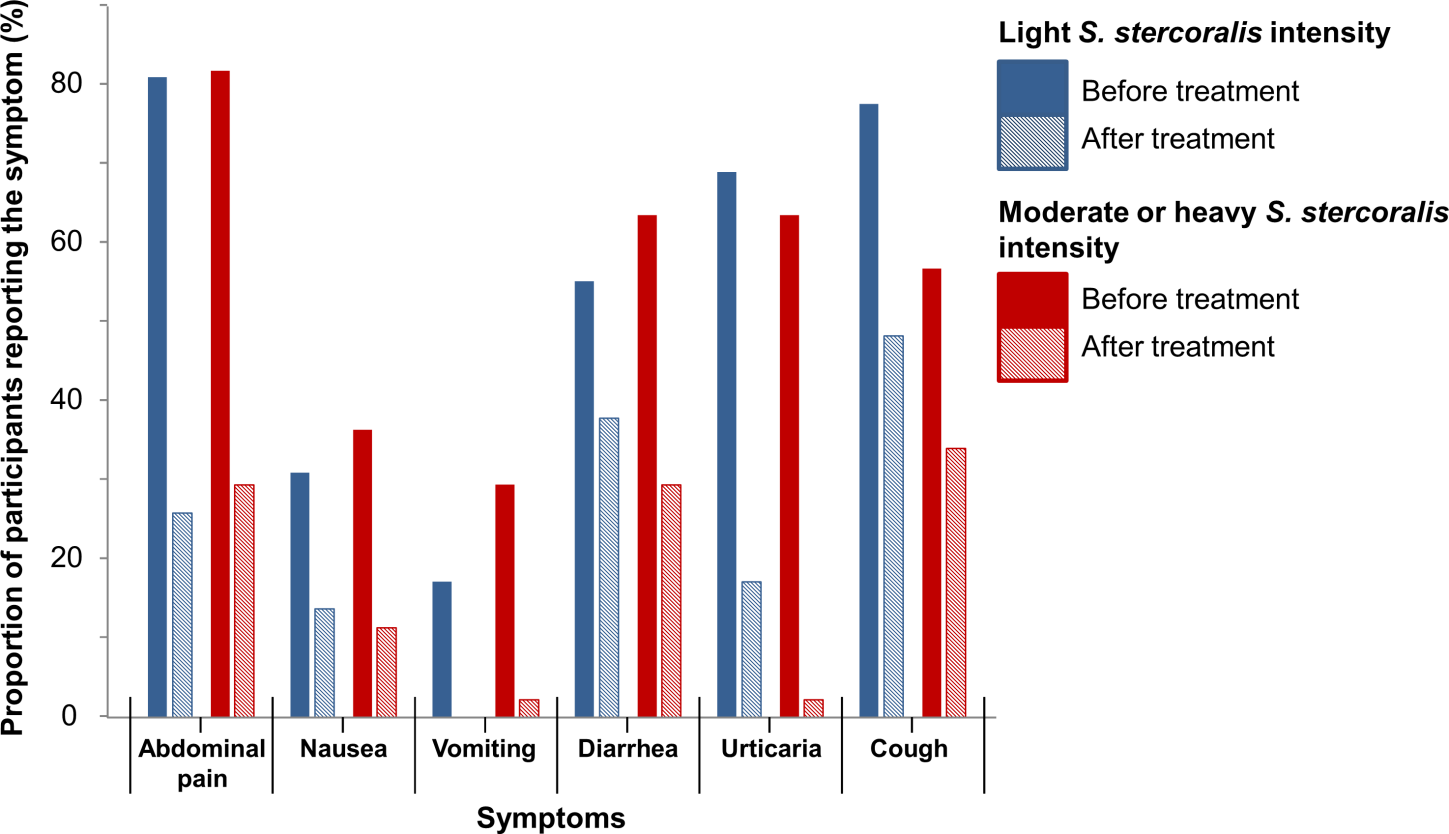
Proportion of participants harboring *S*. *stercoralis* mono-infections and reporting abdominal pain, nausea, vomiting, diarrhea, urticaria, and cough before and 21 days after ivermectin (200 μg/kg BW) treatment. The decreases in the proportion of participants reporting any of the symptoms in the figure was significant at 5% level, as assessed by the McNemar’s test. *S*. *stercoralis* low parasite load: positive count and ≤ 1 larvae per gram (LPG). *S*. *stercoralis* moderate or high parasite load: > 1 LPG. Data were collected in 2012 in Preah Vihear Province, North Cambodia, from 103 participants in the post-treatment survey who harbored *S*. *stercoralis* mono-infection at both surveys and met the case definitions used in this work for all parasites.

Table 3 presents the extent of symptom resolution following single-dose ivermectin treatment among patients with *S*. *stercoralis* mono-infection (i.e. excluding co-infection with any other helminth or pathogenic protozoan parasite). All participants reported at least one symptom before treatment. The symptoms that declined most after treatment were urticaria (66.0% *vs*. 10.7%, OR: 0.03, 95% CI: 0.00–0.13), abdominal pain (80.6% *vs*. 27.2%, OR: 0.07, 95% CI: 0.02–0.18), and vomiting (23.3% *vs*. 1.0%, OR: <0.1, 95% CI: <0.01–0.17). Other symptoms that declined significantly after treatment were nausea, diarrhea, tiredness, and cough. With the addition of 105 patients co-infected with other helminth or pathogenic protozoan parasites, the same symptoms were found to significantly recede post-treatment. Loss of appetite was also less frequently reported in this group (S4 Table). Fig 2 displays the proportions, stratified by parasite load, of the six symptoms that were significantly less frequently reported by the *S*. *stercoralis* patients who were free of any other diagnosed parasite, following treatment. The decreases appear to be of a similar magnitude among participants with light *vs*. moderate or heavy infections.

**Table 3:** Symptoms before and after ivermectin treatment in 103 patients with *S*. *stercoralis* mono-infection.

Symptom	Before treatment	After treatment			
	n (%)	n (%)	OR	95% CI	p-value ^(a)^
Loss of appetite / anorexia	42 (40.8)	30 (29.1)	0.56	0.27–1.08	0.09
Abdominal pain	83 (80.6)	28 (27.2)	**0.07**	0.02–0.18	< 0.001
Nausea	34 (33.0)	13 (12.6)	**0.30**	0.13–0.65	< 0.001
Vomiting	24 (23.3)	1 (1.0)	**< 0.01**	0.00–0.17	< 0.001
Diarrhea	61 (59.2)	35 (34.0)	**0.33**	0.16–0.64	< 0.001
Constipation	12 (11.7)	11 (10.7)	0.89	0.30–2.60	> 0.99
Itching	56 (54.4)	52 (50.5)	0.86	0.48–1.53	0.68
Urticaria	68 (66.0)	11 (10.7)	**0.03**	0.00–0.13	< 0.001
Cough	71 (68.9)	44 (42.7)	**0.29**	0.13–0.58	< 0.001
Wheezing	10 (9.7)	11 (10.7)	1.13	0.39–3.35	> 0.99
Fever	36 (35.0)	45 (43.7)	1.60	0.81–3.28	0.20
Tiredness	35 (34.0)	21 (20.4)	**0.44**	0.20–0.93	0.03
Muscle pain	40 (38.8)	44 (42.7)	1.22	0.63–2.42	0.64

^(a)^ p-values and odds ratios were obtained from McNemar’s exact test.

Treatment: ivermectin 200 μg/kg BW.

OR in bold were significant at 5% level.

Data were obtained from a before-after treatment study conducted in 2012 in Preah Vihear province, Cambodia, among 103 patients with *S*. *stercoralis* mono-infection.

OR: odds ratio.

CI: confidence interval.

### Growth retardation in children and *S*. *stercoralis* infection

More than half of the children were either moderately (40.4%) or severely (11.3%) stunted. The proportion of stunting was 46.6% in *S*. *stercoralis*-infected children and 37.8% in non-infected children. Stunting was associated with *S*. *stercoralis* infection status, with a higher risk of being stunted when infected with *S*. *stercoralis* (OR: 1.35, 95%CI: 1.00–1.81) or when heavily infected compared to uninfected (OR: 2.49, 96%CI: 1.31–4.71). No interactions were found. Results of the association between stunting and *S*. *stercoralis* parasite load are presented in Table 4. Thinness was also common, with one in five children (19.9%) being underweight, but thinness was not associated with either *S*. *stercoralis* infection status (χ^2^ = 0.47, LRT p-value = 0.5) or parasite load (χ^2^ = 1.21, LRT p-value = 0.75).

**Table 4:** Results of the multivariate logistic model assessing the association between stunting and *S*. *stercoralis* parasite load.

		Stunted no	Stunted yes			
Variable	Category	n (%)	n (%)	OR	95%CI	p-value
*S*. *stercoralis* parasite load	No infection	499 (78.6)	304 (72.0)	1.00		
	Light	88 (13.8)	72 (17.1)	1.26	0.89–1.79	0.20
	Moderate	31 (4.9)	20 (4.7)	0.97	0.53–1.76	0.91
	Heavy	17 (2.7)	26 (6.2)	**2.48**	1.31–4.71	0.01
Infected with hookworm	No	495 (77.9)	330 (78.2)	1.00		
	Yes	140 (22.1)	92 (21.8)	0.84	0.61–1.15	0.28
Sex	Male	302 (47.6)	233 (55.2)	1.00		
	Female	333 (52.4)	189 (44.8)	**0.73**	0.57–0.95	0.02
Age (years)	5–9	223 (35.1)	116 (27.5)	1.00		
	10–13	211 (33.2)	153 (36.3)	**1.66**	1.18–2.33	0.003
	14–19	201 (31.7)	153 (36.2)	**1.88**	1.32–2.69	< 0.001
Socioeconomic status	Least poor	267 (42.1)	141 (33.4)	1.00		
	Poor	214 (33.7)	156 (37.0)	**1.44**	1.07–1.94	0.02
	Most Poor	154 (24.2)	125 (29.6)	**1.63**	1.18–2.25	0.003
Occupation	School	548 (86.3)	356 (84.4)	1.00		
	At home	49 (7.7)	41 (9.7)	**1.72**	1.06–2.81	0.03
	Farmer, other	635 (6.0)	422 (5.9)	0.84	0.48–1.46	0.53

Data were obtained from a cross-sectional study conducted in 2012 in Preah Vihear province, Cambodia, among 1,057 children aged 5–19 years.

OR: odds ratio.

CI: confidence interval.

OR in bold were significant at 5% level.

## Discussion

This is the first report, to our knowledge, showing symptoms specifically associated with *S*. *stercoralis*, i.e. excluding other helminth infections and pathogenic protozoa, in a multi-parasitic setting, where it is particularly difficult to assess the morbidity associated with specific STH infections. Both the sensitivity and specificity of helminth diagnosis were maximized by combining all available methods (Baermann, KAP, and Kato-Katz on two stool samples, and FECT on one stool sample), and by including only negatives with the highest “certainty” of negative status, i.e. participants with all four (Baermann and KAP each on two samples) available results for *S*. *stercoralis* and with two (Kato Katz on two samples) results for other helminths. Women and children under six years were more likely to have incomplete diagnostic results for helminths, but this aspect did not bias the analysis as all models were adjusted to account for those factors.

Among the 103 participants infected with *S*. *stercoralis* only, dermatological, gastrointestinal, and respiratory symptoms, such as urticaria, abdominal pain, nausea, vomiting, diarrhea, and cough, were found to be significantly less frequent after treatment. A previous study conducted in the same province also found that abdominal pain, diarrhea, and urticaria were resolved by ivermectin treatment among 21 heavily infected patients [7]. All of those symptoms are consistent with various phases of the infection [5, 6].

The most prominent symptom of *S*. *stercoralis* infection was urticaria, also called “hives”, which was both mostly resolved by ivermectin treatment and associated with infection status. However, the association was weak given the high proportion of *S*. *stercoralis* negative patients also reporting those symptoms and results should be interpreted with caution. The lack of association between reported symptoms and infection status could be due to misclassification of *S*. *stercoralis* cases, however we used a diagnosis approach with sensitivity exceeding 92%, even for light infections, so the number of false negative *S*. *stercoralis* cases would be low [24]. The weak or absent associations between symptoms and *S*. *stercoralis* infection status mostly reflect the difficulty of assessing the relationship between nonspecific clinical signs and STH or protozoan infections in poly-parasite endemic settings [6]. The challenges faced in such assessments tend to include diagnostic approaches with imperfect sensitivity, reported symptoms being subject to recall and reporting biases, and the non-specificity of symptoms that could be due to other pathologies including viral infections.

Treatment had no effect on itching, indicating that itching could be the result of numerous other conditions and reasons, particularly in tropical settings, including allergies and insect bites, and not necessarily be disease-specific [28].

Urticaria is a well-known symptom of chronic strongyloidiasis and has already been identified as such in Cambodia through studies that did not, however, exclude cases of co-infection with other helminth species or pathogenic protozoa [4, 7, 9, 29, 30]. Acute urticaria may occur at the penetration site for hookworms and *S*. *stercoralis* and is mostly located on the feet [8, 9]. Urticaria occurring during the chronic phase of infection is accepted as a systemic reaction due to the parasite-induced immunologic inflammatory response, which results in increased eosinophil and IgE levels, similar to an allergic response [31–33]. However, the actual mechanisms explaining the relationship between skin reactions and helminths remain unclear [33, 34]. Some authors suggest that urticaria is induced by parasites to ease migration under the skin, in the lymphatic ways and in some parenchymatous organs [33]. Therefore, urticaria would relate to the larval stage of infection or to the parasite migration phase, rather than to the mere presence of parasites in the body [33]. This statement is of particular interest to the etiology of urticaria in strongyloidiasis and would be in line with *S*. *stercoralis’* autoinfection ability, whereby the parasite continuously replicates and produces larvae that re-infect the host.

Another striking effect of ivermectin treatment was the resolution of abdominal pain among most of the patients. The impact on other clinical signs—with the exception of vomiting, which was reported by only one patient after treatment—was more modest, as indicated by the substantial proportion of participants still reporting symptoms three weeks after treatment. These symptoms also declined significantly among participants co-infected with *S*. *stercoralis* and other parasites (75% of which were hookworm). Morbidity was not associated with hookworm infection in this study, probably because almost all cases (97.7%) were light intensity infections, as defined by the WHO thresholds, or because of a high prevalence of *Ancylostoma ceylanicum*, a hookworm common to dogs and cats that often infects humans in the region [35–37]. Interestingly, self-reported morbidity was higher among individuals infected with *S*. *stercoralis* than among those with hookworm in a setting endemic for both parasites [6].

Surprisingly, while STH morbidity is known to increase with worm load, the clinical manifestations associated with *S*. *stercoralis* were not associated with parasite loads in this setting. This result could be due to the irregular larval output of *S*. *stercoralis* that might have affected the estimated parasite load. It is also possible that the thresholds used inadequately reflected the impact of parasite load on health or that other undiagnosed pathogenic parasites were effectively treated by ivermectin, thereby having a confounding effect [24, 38–40]. However, confusion between larval output thresholds used for low (≤1 LPG) *vs*. high (≥ 10 LPG) parasite load appears unlikely [24, 38, 39]. Yet, it cannot be excluded that *S*. *stercoralis* is pathogenic even in cases of low parasite load, which would largely affect any indicator of parasite burden or treatment effect, including cost-effectiveness assessments.

An important finding was the association between growth retardation and both *S*. *stercoralis* infection risk and parasite load. The risk of being stunted increased with age, indicating the accumulation of growth retardation through time. This association may also reflect greater exposure to malnutrition in the past, among older children. Stunting may be due to a number of causes, including suffering from heavy STH infections before STH control was implemented. However, in this setting, where about half of the children suffered from growth retardation, the cross-sectional design of this study could not address causality. Further studies accounting for potential confounders of the relationship between malnutrition and *S*. *stercoralis* infection, such as medical history, quantitative and qualitative food intake, and social aspects, are needed to determine specific factors as well as the strength and direction of the association.

Moderate to heavy infections with any of the three other STH, *A*. *lumbricoides*, *T*. *trichiura*, and hookworm, are widely recognized as causes of stunting, which make STH one of the most important causes of physical and intellectual growth impairment [41–44]. Yet, current evidence supporting the association between STH and childhood growth is currently of low quality and warrants further research, which should also include *S*. *stercoralis*. Confirming that *S*. *stercoralis* infection plays a role in growth retardation due to its contribution to chronic malnutrition in childhood would have an important impact on estimating the disease burden.

Protein-calorie malnutrition is a known cause of immunodeficiency in resource-poor countries and may be a pivotal aspect of *S*. *stercoralis* morbidity [2]. First, chronic strongyloidiasis causes gastro-intestinal symptoms that potentially lead to malnutrition through lower food intake and nutrient loss [44]. Second, there is evidence from animal studies that nematode infections in malnourished hosts induce a decrease in the T-Helper Type 2 (Th2) mediated immune response, including eosinophil counts, which are known to be an important part of the immune response against *S*. *stercoralis* [45, 46]. Finally, immunodeficiency may increase the risk of complicated strongyloidiasis in malnourished populations, and malnutrition unrelated to known causes of immunosuppression might be responsible for severe strongyloidiasis cases in developing countries [2, 46, 47]. The risk of developing severe strongyloidiasis could be high in settings with widespread malnutrition such as Cambodia, while an additional issue is the increased availability of over-the-counter drugs containing corticosteroids [2, 48].

Our study has several limitations. The difference in durations considered for symptom reporting before and after treatment may have overestimated pre-treatment symptom reporting frequencies as well as treatment effects, particularly in the case of vomiting. Additionally, in the absence of a control group, our study did not account for placebo effects, which could have influenced symptom reporting.

Finally, some co-infections with pathogenic protozoa may have been missed due to the limited sensitivity of FECT performed on one stool sample, which might explain the moderate impact of ivermectin treatment on diarrhea and nausea. However, this limitation would not apply to helminths, for which the diagnostic approach used in the present study has been assessed several times and has shown high sensitivity and specificity [17, 23, 24, 29]. Nor would it apply to urticaria, which is commonly associated with other helminths including *Ascaris lumbricoides*, *Hymenolepis nana*, and *Fasciola hepatica*, as well as with protozoans including *Giardia lamblia* and *Blastocystis hominis* [28, 30, 33, 49, 50]. While we cannot exclude that some protozoan infections were missed, ivermectin is not effective against *G*. *lamblia* or *B*. *hominis*, and so the resolution of urticaria, a widely recognized symptom of chronic strongyloidiasis, in 84% of participants in the before-after study would appear to arise from clearance of *S*. *stercoralis*. Scabies is another important cause of itching/urticaria in developing countries that would be resolved by ivermectin treatment [51]. However, the basic medical examination that was conducted during data collection included a skin check, which would have led to scabies diagnosis if present.

Our combined results demonstrate that the burden of strongyloidiasis, which encompasses all health states from mild symptoms to severe, life-threatening infection, might be much higher in endemic settings than previously thought. Chronic strongyloidiasis appears to cause both acute gastrointestinal symptoms and urticaria, all bothersome symptoms that are experienced whatever the age of the individual and intensity of infection. It also causes subtle long-term health effects through its association with malnutrition.

Next steps towards estimating the *S*. *stercoralis* burden include assessing the extent of strongyloidiasis morbidity, including growth retardation and malnutrition, with regard to infection intensity—for which standards have yet to be established, and estimating the risk of severe strongyloidiasis and hyperinfection in endemic settings. *S*. *stercoralis* is not currently addressed by the WHO control strategy against STH that relies on “preventive chemotherapy”, i.e. regular mebendazole or albendazole treatment of specific at-risk groups or mass-drug administration (MDA) [52, 53]. Single dose benzimidazoles have suboptimal effects on *S*. *stercoralis*, for which ivermectin is the drug of choice. This drug is highly efficacious at a single oral dose of 200 μg/kg body weight (BW) and is well tolerated. Moreover, ivermectin is also efficacious against *Ascaris lumbricoides*. In combination with benzimidazoles, it improves therapeutic outcomes against *Trichuris trichiura*, while in combination with albendazole, it improves therapeutic performances against hookworm [14–16, 54, 55].

In the absence of infection intensity figures, the similarity of reinfection rates and morbidity across age groups support arguments for community-wide control [13]. However, the long-term impact of malnutrition on childhood development could justify integrating *S*. *stercoralis* control into ongoing school-based STH control programs, which are well established throughout the country and currently target children from infancy to high school. Monitoring the impact of control on infection levels in various transmission settings would help to assess whether, where and how control measures should be extended, while optimizing cost-effectiveness. The cost of ivermectin poses a challenge to expanding its use. In Cambodia, the drug is neither donated nor subsidized and treating one individual with quality tablets produced by a certified manufacturing company costs 20 to 40 USD, depending on the patient’s weight. The high prevalence of the parasite and its significant morbidity clearly advocate for increased donations or the production of generic ivermectin so *S*. *stercoralis* control can be implemented without further delay in Cambodia.

## Supporting information

S1 Strobe checklist (PDF)

S1 Table Prevalences and number of cases of all diagnosed helminths and protozoa. Data were obtained from a cross-sectional survey carried out 2012 in eight villages of Preah Vihear province, Cambodia, among 2,744 participants meeting the study case definition for all diagnosed parasites. ^a^ non-pathogenic. ^b^ pathogenic. n: number of cases; CI: confidence interval.
(PDF)

S2 Table Association between symptoms and *S*. *stercoralis* parasite load. *S*. *stercoralis* parasite load: positive count and ≤ 1 larvae per gram (LPG). *S*. *stercoralis* moderate or heavy parasite load: > 1 LPG. ^(a)^ Odds ratios were adjusted for sex, age, treatment and infection with any diagnosed helminth or pathogenic protozoa. Treatment corresponds to uptake of anthelmintic tablets within the past year. Data were obtained from a cross-sectional survey carried out 2012 in eight villages of Preah Vihear province, Cambodia, among 2,612 participants with *S*. *stercoralis* infection intensity data. OR: odds ratio; CI: confidence interval; LRT: likelihood ratio test.
(PDF)

S3 Table Complete results of multivariate logistic regressions assessing the association between each reported symptom and *S*. *stercoralis* infection status. Data were obtained from 2,744 participants in a cross-sectional survey carried out 2012 in eight villages of Preah Vihear province, Cambodia. OR: odds ratio; CI: confidence interval.
(PDF)

S4 Table Symptoms reported before and after ivermectin treatment by *S*. *stercoralis* infected patients including co-infection with other parasites (208 patients). Treatment: ivermectin 200 μg/kg BW. OR in bold were significant at 95% level. LRT p-values were obtained from conditional logistic regressions. Data were collected in 2012 in 2 villages of Preah Vihear Province, North Cambodia, from 208 *S*. *stercoralis* patients regardless of infection with any other diagnosed helminth or protozoa and met the cases definitions used in this work for all parasites. The total of 208 patients is constituted of 103 patients free of any infection other than *S*. *stercoralis* and 105 patients co-infected by any other parasite at any survey. OR: odds ratio; CI: confidence interval; LRT: likelihood ratio test.
(PDF)

## References

[1] Schär F, Trostdorf U, Giardina F, Khieu V, Muth S, Marti H, et al *Strongyloides stercoralis*: Global Distribution and Risk Factors. PLoS Negl Trop Dis. 2013;7(7). 10.1371/journal.pntd.0002288 PMC3708837 23875033

[2] Olsen A, van Lieshout L, Marti H, Polderman T, Polman K, Steinmann P, et al Strongyloidiasis—the most neglected of the neglected tropical diseases? Trans R Soc Trop Med Hyg. 2009;103(10):967–72. doi: 10.1016/j.trstmh.2009.02.013 . 19328508 10.1016/j.trstmh.2009.02.013

[3] Bisoffi Z, Buonfrate D, Montresor A, Requena-Méndez A, Muñoz J, Krolewiecki AJ, et al *Strongyloides stercoralis*: a plea for action. PLoS Negl Trop Dis. 2013;7(5):e2214 doi: 10.1371/journal.pntd.0002214 ; PubMed Central PMCID: PMC3649953. 23675546 10.1371/journal.pntd.0002214 PMC3649953

[4] Krolewiecki AJ, Lammie P, Jacobson J, Gabrielli AF, Levecke B, Socias E, et al A public health response against *Strongyloides stercoralis*: time to look at soil-transmitted helminthiasis in full. PLoS Negl Trop Dis. 2013;7(5):e2165 doi: 10.1371/journal.pntd.0002165 ; PubMed Central PMCID: PMC3649958. 23675541 10.1371/journal.pntd.0002165 PMC3649958

[5] Grove DI. Human strongyloidiasis. Adv Parasitol. 1996;38:251–309. . 8701797 10.1016/s0065-308x(08)60036-6

[6] Becker SL, Sieto B, Silue KD, Adjossan L, Kone S, Hatz C, et al Diagnosis, clinical features, and self-reported morbidity of *Strongyloides stercoralis* and hookworm infection in a co-endemic setting. PLoS Negl Trop Dis. 2011;5(8):e1292 doi: 10.1371/journal.pntd.0001292 ; PubMed Central PMCID: PMC3160297. 21886853 10.1371/journal.pntd.0001292 PMC3160297

[7] Khieu V, Srey S, Schär F, Muth S, Marti H, Odermatt P. *Strongyloides stercoralis* is a cause of abdominal pain, diarrhea and urticaria in rural Cambodia. BMC Res Notes. 2013;6. 10.1186/1756-0500-6-200 PMC3668207 23688049

[8] Toledo R, Muñoz-Antoli C, Esteban JG. Strongyloidiasis with emphasis on human infections and its different clinical forms. Adv Parasitol. 2015;88:165–241. doi: 10.1016/bs.apar.2015.02.005 . 25911368 10.1016/bs.apar.2015.02.005

[9] Nutman TB. Human infection with *Strongyloides stercoralis* and other related *Strongyloides* species. Parasitology. 2016:1–11. doi: 10.1017/S0031182016000834 . 27181117 10.1017/S0031182016000834 PMC5563389

[10] Keiser PB, Nutman TB. *Strongyloides stercoralis* in the Immunocompromised Population. Clin Microbiol Rev. 2004;17(1):208–17. doi: 10.1128/CMR.17.1.208-217.2004 ; PubMed Central PMCID: PMC321465. 14726461 10.1128/CMR.17.1.208-217.2004 PMC321465

[11] Henriquez-Camacho C, Gotuzzo E, Echevarria J, White AC Jr., Terashima A, Samalvides F, et al Ivermectin versus albendazole or thiabendazole for *Strongyloides stercoralis* infection. The Cochrane database of systematic reviews. 2016;1:CD007745 doi: 10.1002/14651858.CD007745.pub3 . 26778150 10.1002/14651858.CD007745.pub3 PMC4916931

[12] Barda B, Sayasone S, Phongluxa K, Xayavong S, Keoduangsy K, Odermatt P, et al Efficacy of moxidectin versus ivermectin against *Strongyloides stercoralis* infections: a randomized controlled non-inferiority trial. Clinical infectious diseases: an official publication of the Infectious Diseases Society of America. 2017 doi: 10.1093/cid/cix278 . 28369530 10.1093/cid/cix278

[13] Forrer A, Khieu V, Schindler C, Schär F, Marti H, Char MC, et al Ivermectin Treatment and Sanitation Effectively Reduce *Strongyloides stercoralis* Infection Risk in Rural Communities in Cambodia. PLoS Negl Trop Dis. 2016;10(8):e0004909 doi: 10.1371/journal.pntd.0004909 . 27548286 10.1371/journal.pntd.0004909 PMC4993485

[14] Igual-Adell R, Oltra-Alcaraz C, Soler-Company E, Sánchez-Sánchez P, Matogo-Oyana J, Rodríguez-Calabuig D. Efficacy and safety of ivermectin and thiabendazole in the treatment of strongyloidiasis. Expert Opin Pharmacother. 2004;5(12):2615–9. doi: 10.1517/14656566.5.12.2615 15571478 10.1517/14656566.5.12.2615

[15] Suputtamongkol Y, Premasathian N, Bhumimuang K, Waywa D, Nilganuwong S, Karuphong E, et al Efficacy and safety of single and double doses of ivermectin versus 7-day high dose albendazole for chronic strongyloidiasis. PLoS Negl Trop Dis. 2011;5(5):e1044 doi: 10.1371/journal.pntd.0001044 ; PubMed Central PMCID: PMC3091835. 21572981 10.1371/journal.pntd.0001044 PMC3091835

[16] Gann PH, Neva FA, Gam AA. A randomized trial of single- and two-dose ivermectin versus thiabendazole for treatment of strongyloidiasis. J Infect Dis. 1994;169(5):1076–9. . 8169394 10.1093/infdis/169.5.1076

[17] Khieu V, Schär F, Forrer A, Hattendorf J, Marti H, Duong S, et al High prevalence and spatial distribution of *Strongyloides stercoralis* in rural Cambodia. PLoS Negl Trop Dis. 2014;8(6):e2854 doi: 10.1371/journal.pntd.0002854 ; PubMed Central PMCID: PMC4055527. 24921627 10.1371/journal.pntd.0002854 PMC4055527

[18] National Center for Parasitology EaMC, Ministry of Health, Phnom Penh, Cambodia. National Policy and Guideline for Helminth Control in Cambodia. 2004. p. 44p.

[19] Koga K, Kasuya S, Khamboonruang C, Sukhavat K, Ieda M, Takatsuka N, et al A modified agar plate method for detection of *Strongyloides stercoralis*. Am J Trop Med Hyg. 1991;45(4):518–21. . 1951861 10.4269/ajtmh.1991.45.518

[20] Baermann G. A simple method for the detection of Ankylostomum (nematode) larvae in soil tests. 1917:41–7.

[21] Katz N, Chaves A, Pellegrino J. A simple device for quantitative stool thick-smear technique in *Schistosomiasis mansoni*. Rev Inst Med Trop Sao Paulo. 1972;14(6):397–400. . 4675644

[22] Marti H, Escher E. SAF—an alternative fixation solution for parasitological stool specimens. Schweiz Med Wochenschr. 1990;120(40):1473–6. 2218469

[23] Khieu V, Schär F, Marti H, Sayasone S, Duong S, Muth S, et al Diagnosis, treatment and risk factors of *Strongyloides stercoralis* in schoolchildren in Cambodia. PLoS Negl Trop Dis. 2013;7(2). 10.1371/journal.pntd.0002035 PMC3566990 23409200

[24] Schär F, Hattendorf J, Khieu V, Muth S, Char MC, Marti HP, et al *Strongyloides stercoralis* larvae excretion patterns before and after treatment. Parasitology. 2014:1–6. doi: 10.1017/S0031182013002345 . 24534076 10.1017/S0031182013002345

[25] RCoreTeam. R: A language and environment for statistical computing. R Foundation for Statistical Computing, Vienna, Austria. 2016.

[26] de Onis M OA, Borghi E, Siyam A, Nishida C, Siekmann J. Development of a WHO growth reference for school-aged children and adolescents. BullWorld Health Organ. 2007;85(9):649–732. 10.2471/BLT.07.043497 PMC2636412 18026621

[27] Group WMGR. Child Growth Standards: Length/height-for-age, weight-for-age, weight-for-length, weight-for-height and body mass index-for-age: methods and development. 2006.

[28] Caraballo L, Zakzuk J, Lee BW, Acevedo N, Soh JY, Sánchez-Borges M, et al Particularities of allergy in the Tropics. World Allergy Organ J. 2016;9:20 doi: 10.1186/s40413-016-0110-7 ; PubMed Central PMCID: PMCPMC4924335. 27386040 10.1186/s40413-016-0110-7 PMC4924335

[29] Khieu V, Schär F, Marti H, Bless PJ, Char MC, Muth S, et al Prevalence and risk factors of *Strongyloides stercoralis* in Takeo Province, Cambodia. Parasit Vectors. 2014;7:221 doi: 10.1186/1756-3305-7-221 ; PubMed Central PMCID: PMCPMC4029906. 24886763 10.1186/1756-3305-7-221 PMC4029906

[30] Kolkhir P, Balakirski G, Merk HF, Olisova O, Maurer M. Chronic spontaneous urticaria and internal parasites—a systematic review. Allergy. 2016;71(3):308–22. doi: 10.1111/all.12818 . 26648083 10.1111/all.12818

[31] Rampur L, Jariwala SP, Hudes G, Rosenstreich DL, de Vos G. Effect of ivermectin on allergy-type manifestations in occult strongyloidiasis. Ann Allergy Asthma Immunol. 2016;117(4):423–8. doi: 10.1016/j.anai.2016.07.021 . 27566864 10.1016/j.anai.2016.07.021

[32] Sanders NL, Mishra A. Role of interleukin-18 in the pathophysiology of allergic diseases. Cytokine Growth Factor Rev. 2016;32:31–9. doi: 10.1016/j.cytogfr.2016.07.001 ; PubMed Central PMCID: PMCPMC5124539. 27496752 10.1016/j.cytogfr.2016.07.001 PMC5124539

[33] Bakiri AH, Mingomataj EC. Parasites induced skin allergy: a strategic manipulation of the host immunity. J Clin Med Res. 2010;2(6):247–55. doi: 10.4021/jocmr456w ; PubMed Central PMCID: PMCPMC3194028. 22043257 10.4021/jocmr456w PMC3194028

[34] Nahshoni A, Baum S, Barzilai A, Schwartz E. Chronic Urticaria in Returning Travellers: The Role of Anthelmintic Treatment. Dermatology. 2016;232(4):468–71. doi: 10.1159/000445715 . 27246602 10.1159/000445715

[35] Inpankaew T, Schär F, Dalsgaard A, Khieu V, Chimnoi W, Chhoun C, et al High prevalence of *Ancylostoma ceylanicum* hookworm infections in humans, Cambodia, 2012. Emerg Infect Dis. 2014;20(6):976–82. doi: 10.3201/eid2006.131770 ; PubMed Central PMCID: PMC4036766. 24865815 10.3201/eid2006.131770 PMC4036766

[36] Traub RJ. *Ancylostoma ceylanicum*, a re-emerging but neglected parasitic zoonosis. Int J Parasitol. 2013;43(12–13):1009–15. doi: 10.1016/j.ijpara.2013.07.006 . 23968813 10.1016/j.ijpara.2013.07.006

[37] WHO. Prevention and control of schistosomiasis and soil-transmitted helminthiasis: a report of a WHO expert committee 2002. 1–57]. 12592987

[38] Uparanukraw P, Phongsri S, Morakote N. Fluctuations of larval excretion in *Strongyloides stercoralis* infection. Am J Trop Med Hyg. 1999;60(6):967–73. . 10403329 10.4269/ajtmh.1999.60.967

[39] Sato Y, Kobayashi J, Toma H, Shiroma Y. Efficacy of stool examination for detection of *Strongyloides* infection. Am J Trop Med Hyg. 1995;53(3):248–50. . 7573706 10.4269/ajtmh.1995.53.248

[40] Siddiqui AA, Berk SL. Diagnosis of *Strongyloides stercoralis* infection. Clinical infectious diseases: an official publication of the Infectious Diseases Society of America. 2001;33(7):1040–7. doi: 10.1086/322707 . 11528578 10.1086/322707

[41] Hotez P, de Silva N, Brooker S, Bethony J. Soil Transmitted Helminth Infections: the nature, causes and burden of the condition. Bethesda, Maryland, USA: Fogarty International Center, National Institutes of Health, 2003.

[42] Yap P, Utzinger J, Hattendorf J, Steinmann P. Influence of nutrition on infection and re-infection with soil-transmitted helminths: a systematic review. Parasit Vectors. 2014;7:229 doi: 10.1186/1756-3305-7-229 ; PubMed Central PMCID: PMC4032457. 24885622 10.1186/1756-3305-7-229 PMC4032457

[43] Bethony J, Brooker S, Albonico M, Geiger SM, Loukas A, Diemert D, et al Soil-transmitted helminth infections: ascariasis, trichuriasis, and hookworm. Lancet. 2006;367(9521):1521–32. doi: 10.1016/S0140-6736(06)68653-4 16679166 10.1016/S0140-6736(06)68653-4

[44] Stephenson LS, Latham MC, Ottesen EA. Malnutrition and parasitic helminth infections. Parasitology. 2000;121 Suppl:S23–38. . 11386688 10.1017/s0031182000006491

[45] Koski KG, Scott ME. Gastrointestinal nematodes, nutrition and immunity: breaking the negative spiral. Annu Rev Nutr. 2001;21:297–321. doi: 10.1146/annurev.nutr.21.1.297 . 11375439 10.1146/annurev.nutr.21.1.297

[46] Concha R, Harrington W Jr., Rogers AI. Intestinal strongyloidiasis: recognition, management, and determinants of outcome. J Clin Gastroenterol. 2005;39(3):203–11. . 15718861 10.1097/01.mcg.0000152779.68900.33

[47] Cimino RO, Krolewiecki A. The Epidemiology of Human Strongyloidiasis. Current Tropical Medicine Reports. 2014;1(4):216–22.

[48] Greffeuille V, Sophonneary P, Laillou A, Gauthier L, Hong R, Hong R, et al Persistent Inequalities in Child Undernutrition in Cambodia from 2000 until Today. Nutrients. 2016;8(5). doi: 10.3390/nu8050297 ; PubMed Central PMCID: PMCPMC4882710. 27196924 10.3390/nu8050297 PMC4882710

[49] Lepczynska M, Chen WC, Dzika E. Mysterious chronic urticaria caused by *Blastocystis* spp.? Int J Dermatol. 2015 doi: 10.1111/ijd.13064 . 26469206 10.1111/ijd.13064

[50] Giacometti A, Cirioni O, Antonicelli L, D'Amato G, Silvestri C, Del Prete MS, et al Prevalence of intestinal parasites among individuals with allergic skin diseases. J Parasitol. 2003;89(3):490–2. doi: 10.1645/0022-3395(2003)089[0490:POIPAI]2.0.CO;2 . 12880246 10.1645/0022-3395(2003)089[0490:POIPAI]2.0.CO;2

[51] Hay RJ, Steer AC, Engelman D, Walton S. Scabies in the developing world—its prevalence, complications, and management. Clinical microbiology and infection: the official publication of the European Society of Clinical Microbiology and Infectious Diseases. 2012;18(4):313–23. doi: 10.1111/j.1469-0691.2012.03798.x . 22429456 10.1111/j.1469-0691.2012.03798.x

[52] WHO, Crompton DW. Preventive chemotherapy in human helminthiasis: coordinated use of anthelminthic drugs in control interventions: a manual for health professionals and programme managers. Geneva: World Health Organization 2006. 62 p.

[53] WHO. First WHO report on neglected tropical diseases: "Working to overcome the global impact of negleted tropical diseases" Geneva: World Health Organization 2010.

[54] Keiser J, Utzinger J. The drugs we have and the drugs we need against major helminth infections. Adv Parasitol. 2010;73:197–230. doi: 10.1016/S0065-308X(10)73008-6 . 20627144 10.1016/S0065-308X(10)73008-6

[55] Knopp S, Mohammed KA, Speich B, Hattendorf J, Khamis IS, Khamis AN, et al Albendazole and mebendazole administered alone or in combination with ivermectin against *Trichuris trichiura*: a randomized controlled trial. Clinical infectious diseases: an official publication of the Infectious Diseases Society of America. 2010;51(12):1420–8. doi: 10.1086/657310 . 21062129 10.1086/657310

