# Supplementary material for: Strongyloides stercoralis is associated with significant morbidity in rural Cambodia, including stunting in children
Source: PLoS Negl Trop Dis. 2017 Oct 23;11(10):e0005685. doi: 10.1371/journal.pntd.0005685 (PMC5695629; doi:10.1371/journal.pntd.0005685)
Supplement: S1 Table — Data were obtained from a cross-sectional survey carried out 2012 in eight villages of Preah Vihear province, Cambodia, among 2,744 participants meeting the study case definition for all diagnosed parasites. a non-pathogenic. b pathogenic. n: number of cases; CI: confidence interval. (PDF) [file pntd.0005685.s002.pdf]

S1 Table. Prevalences and number of cases of all diagnosed helminths and protozoa

**Helminths**	n	Prevalence (%)	95% CI
Strongyloides stercoralis	853	31.09	29.36-32.86
Hookworm	699	25.47	23.85-27.15
Fasciola/Fasciolopsis	85	3.10	2.48-3.82
Opisthorchis viverrini	43	1.57	1.14-2.11
Taenia spp.	22	0.80	0.50-1.21
Dicrocoelium dendriticum	10	0.36	0.17-0.67
Enterobius vermicularis	8	0.29	0.13-0.57
Hymenolepis nana	6	0.22	0.08-0.48
Trichuris trichiura	2	0.07	0.01-0.26
Clonorchis sinensis	1	0.04	<0.01-0.20
**Protozoa**	n	Prevalence (%)	95% CI
Entoamoeba coli [a]	632	23.03	21.47-24.65
Blastocystis hominis [a]	504	18.37	16.93-19.87
Giardia lamblia [b]	198	7.22	6.28-8.25
Iodamoeba bütschlii [a]	135	4.92	4.14-5.80
Endolimax nana [a]	112	4.08	3.37-4.89
Entamoeba histolytica/dispar [b]	32	1.17	0.80-1.64
Entamoeba hartmanni [a]	12	0.44	0.23-0.76
Chilomastix mesnili [a]	2	0.07	0.01-0.26
Sarcocystis spp. [b]	1	0.04	0.0004-0.20

Data were obtained from a cross-sectional survey carried out 2012 in eight villages of Preah Vihear province, Cambodia, among 2,744 participants meeting the study case definition for all diagnosed parasites.

[a] non-pathogenic.

[b] pathogenic.

n: number of cases; CI: confidence interval**.**
