# Supplementary material for: Strongyloides stercoralis is associated with significant morbidity in rural Cambodia, including stunting in children
Source: PLoS Negl Trop Dis. 2017 Oct 23;11(10):e0005685. doi: 10.1371/journal.pntd.0005685 (PMC5695629; doi:10.1371/journal.pntd.0005685)
Supplement: S2 Table — S. stercoralis parasite load: positive count and ≤ 1 larvae per gram (LPG). S. stercoralis moderate or heavy parasite load: > 1 LPG. (a) Odds ratios were adjusted for sex, age, treatment and infection with any diagnosed helminth or pathogenic protozoa. Treatment corresponds to uptake of anthelmintic tablets within the past year. Data were obtained from a cross-sectional survey carried out 2012 in eight villages of Preah Vihear province, Cambodia, among 2,612 participants with S. stercoralis infection intensity data. OR: odds ratio; CI: confidence interval; LRT: likelihood ratio test. (PDF) [file pntd.0005685.s003.pdf]

S2 Table. Association between symptoms and *S. stercoralis* parasite load

Symptom	*S. stercoralis* parasite load	Symptom present n (%)	OR [(a)]	95% CI	LRT p-value
Loss of appetite / Anorexia	No infection	598 (31.6)	1.00		
	Light	149 (34.3)	1.08	0.85 - 1.38	0.381
	Moderate or Heavy	87 (30.3)	0.85	0.63 - 1.14	
Nausea	No infection	611 (32.3)	1.00		
	Light	147 (33.9)	1.02	0.81 - 1.28	0.595
	Moderate or Heavy	89 (31.0)	0.88	0.66 - 1.15	
Vomiting	No infection	380 (20.1)	1.00		
	Light	94 (21.7)	1.09	0.84 - 1.42	0.805
	Moderate or Heavy	60 (20.9)	1.04	0.76 - 1.43	
Abdominal pain	No infection	1,479 (78.2)	1.00		
	Light	358 (82.5)	1.10	0.82 - 1.49	0.632
	Moderate or Heavy	242 (84.3)	1.16	0.80 - 1.67	
Epigastric pain	No infection	1,085 (57.4)	1.00		
	Light	264 (60.8)	0.94	0.74 - 1.21	0.867
	Moderate or Heavy	185 (64.5)	1.03	0.76 - 1.39	
Diarrhea	No infection	1,086 (57.4)	1.00		
	Light	260 (59.9)	1.08	0.86 - 1.34	0.647
	Moderate or Heavy	172 (59.9)	1.11	0.85 - 1.44	
Constipation	No infection	293 (15.5)	1.00		
	Light	60 (13.8)	0.84	0.62 - 1.15	0.531
	Moderate or Heavy	44 (15.3)	0.91	0.64 - 1.31	
Cough	No infection	1,208 (63.9)	1.00		
	Light	277 (63.8)	1.05	0.84 - 1.31	0.563
	Moderate or Heavy	170 (59.2)	0.89	0.69 - 1.16	
Wheezing	No infection	147 (7.8)	1.00		
	Light	38 (8.8)	1.07	0.73 - 1.57	0.879
	Moderate or Heavy	23 (8.0)	0.93	0.58 - 1.49	
Itching	No infection	902 (47.7)	1.00		
	Light	220 (50.7)	1.11	0.90 - 1.38	0.149
	Moderate or Heavy	155 (54.0)	1.27	0.98 - 1.64	
Urticaria	No infection	880 (46.5)	1.00		
	Light	240 (55.3)	1.35	1.08 - 1.68	0.003
	Moderate or Heavy	163 (56.8)	1.41	1.09 - 1.83	
Generalized rash	No infection	345 (18.2)	1.00		
	Light	104 (24.0)	1.36	1.05 - 1.76	0.075
	Moderate or Heavy	58 (19.4)	1.06	0.77 - 1.46	
Fever	No infection	855 (45.2)	1.00		
	Light	192 (44.2)	1.06	0.85 - 1.32	0.499
	Moderate or Heavy	111 (38.7)	0.88	0.68 - 1.15	

Tiredness	No infection	421 (22.3)	1.00		
	Light	109 (25.1)	1.13	0.86 - 1.48	0.644
	Moderate or Heavy	67 (23.3)	0.98	0.71 - 1.35	
Muscle pain	No infection	688 (36.4)	1.00		
	Light	195 (44.9)	1.21	0.96 - 1.53	0.268
	Moderate or Heavy	125 (43.6)	1.06	0.81 - 1.40	

S. stercoralis light parasite load: positive count and ≤ 1 larvae per gram (LPG). *S. stercoralis* moderate or heavy parasite load: > 1 LPG.

[(a)] Odds ratios were adjusted for sex, age, treatment and infection with any diagnosed helminth or pathogenic protozoa.

Treatment corresponds to uptake of anthelmintic tablets within the past year.

Data were obtained from a cross-sectional survey carried out 2012 in eight villages of Preah Vihear province, Cambodia, among 2,612 participants with *S. stercoralis* infection intensity data.

OR: odds ratio; CI: confidence interval; LRT: likelihood ratio test.
