# Supplementary material for: Strongyloides stercoralis is associated with significant morbidity in rural Cambodia, including stunting in children
Source: PLoS Negl Trop Dis. 2017 Oct 23;11(10):e0005685. doi: 10.1371/journal.pntd.0005685 (PMC5695629; doi:10.1371/journal.pntd.0005685)
Supplement: S3 Table — Data were obtained from 2,744 participants in a cross-sectional survey carried out 2012 in eight villages of Preah Vihear province, Cambodia. OR: odds ratio; CI: confidence interval. (PDF) [file pntd.0005685.s004.pdf]

S3 Table. Complete results of multivariate logistic regressions assessing the association between each reported symptom and *S. stercoralis* infection risk

Loss of appetite/anorexia

Variable	Category	OR	95%CI	p-value
S. stercoralis infection	No	1.00		
	Yes	0.97	0.80 - 1.17	0.731
Hookworm infection	No	1.00		
	Yes	0.86	0.70 - 1.06	0.153
Other helminth infection	No	1.00		
	Yes	1.13	0.79 - 1.61	0.497
Pathogenic protozoa infection	No	1.00		
	Yes	0.96	0.68 - 1.36	0.833
Sex	Male	1.00		
	Female	1.40	1.16 - 1.68	<0.0001
Age (years)	6 - 18	1.00		
	< 6	0.80	0.51 - 1.28	0.358
	19 - 59	4.54	3.66 - 5.63	<0.0001
	≥ 60	16.35	11.17 - 23.94	<0.0001
Anthelmintic treatment, past year	No	1.00		
	Yes	1.23	1.02 - 1.48	0.034

Nausea

Variable	Category	OR	95%CI	p-value
S. stercoralis infection	No	1.00		
	Yes	1.02	0.85 - 1.22	0.855
Hookworm infection	No	1.00		
	Yes	1.08	0.90 - 1.31	0.409
Other helminth infection	No	1.00		
	Yes	1.05	0.76 - 1.46	0.756
Pathogenic protozoa infection	No	1.00		
	Yes	0.90	0.67 - 1.22	0.512
Sex	Male	1.00		
	Female	0.97	0.82 - 1.14	0.693
Age (years)	6 - 18	1.00		
	< 6	0.94	0.67 - 1.33	0.728
	19 - 59	1.73	1.44 - 2.07	<0.0001
	≥ 60	1.05	0.73 - 1.51	0.795
Anthelmintic treatment, past year	No	1.00		
	Yes	1.32	1.11 - 1.58	0.002

Vomiting

Variable	Category	OR	95%CI	p-value
S. stercoralis infection	No	1.00		
	Yes	1.05	0.85 - 1.29	0.670
Hookworm infection	No	1.00		
	Yes	1.10	0.89 - 1.37	0.384
Other helminth infection	No	1.00		
	Yes	1.00	0.68 - 1.47	0.992
Pathogenic protozoa infection	No	1.00		
	Yes	1.01	0.72 - 1.41	0.962
Sex	Male	1.00		
	Female	0.90	0.74 - 1.10	0.301
Age (years)	6 - 18	1.00		
	< 6	1.00	0.69 - 1.45	0.988
	19 - 59	1.10	0.89 - 1.35	0.379
	≥ 60	0.47	0.28 - 0.79	0.004
Anthelmintic treatment, past year	No	1.00		
	Yes	1.33	1.08 - 1.63	0.006

Abdominal pain

Variable	Category	OR	95%CI	p-value
S. stercoralis infection	No	1.00		
	Yes	1.11	0.88 - 1.41	0.364
Hookworm infection	No	1.00		
	Yes	0.95	0.74 - 1.21	0.669
Other helminth infection	No	1.00		
	Yes	1.42	0.92 - 2.21	0.116
Pathogenic protozoa infection	No	1.00		
	Yes	0.97	0.69 - 1.35	0.844
Sex	Male	1.00		
	Female	0.92	0.75 - 1.13	0.440
Age (years)	6 - 18	1.00		
	< 6	0.28	0.20 - 0.38	< 0.001
	19 - 59	5.32	4.17 - 6.79	< 0.001
	≥ 60	3.41	2.15 - 5.42	< 0.001
Anthelmintic treatment, past year	No	1.00		
	Yes	1.70	1.37 - 2.11	< 0.001

Epigastric pain

Variable	Category	OR	95%CI	p-value
S. stercoralis infection	No	1.00		
	Yes	0.98	0.81 - 1.20	0.850
Hookworm infection	No	1.00		
	Yes	0.97	0.79 - 1.20	0.800
Other helminth infection	No	1.00		
	Yes	1.30	0.91 - 1.85	0.154
Pathogenic protozoa infection	No	1.00		
	Yes	0.93	0.68 - 1.27	0.629
Sex	Male	1.00		
	Female	1.08	0.90 - 1.30	0.401
Age (years)	6 - 18	1.00		
	< 6	0.18	0.11 - 0.29	< 0.001
	19 - 59	7.11	5.85 - 8.63	< 0.001
	≥ 60	7.09	4.81 - 10.44	< 0.001
Anthelmintic treatment, past year	No	1.00		
	Yes	1.63	1.34 - 1.97	< 0.001

Diarrhea

Variable	Category	OR	95%CI	p-value
S. stercoralis infection	No	1.00		
	Yes	1.10	0.93 - 1.31	0.276
Hookworm infection	No	1.00		
	Yes	0.96	0.80 - 1.15	0.673
Other helminth infection	No	1.00		
	Yes	0.96	0.70 - 1.32	0.816
Pathogenic protozoa infection	No	1.00		
	Yes	1.11	0.83 - 1.47	0.479
Sex	Male	1.00		
	Female	0.69	0.59 - 0.82	< 0.001
Age (years)	6 - 18	1.00		
	< 6	1.09	0.79 - 1.49	0.613
	19 - 59	1.07	0.90 - 1.27	0.468
	≥ 60	0.69	0.50 - 0.96	0.027
Anthelmintic treatment, past year	No	1.00		
	Yes	1.82	1.54 - 2.15	< 0.0001

Constipation

Variable	Category	OR	95%CI	p-value
S. stercoralis infection	No	1.00		
	Yes	0.91	0.71 - 1.15	0.422
Hookworm infection	No	1.00		
	Yes	0.99	0.77 - 1.27	0.93
Other helminth infection	No	1.00		
	Yes	1.33	0.89 - 2.00	0.168
Pathogenic protozoa infection	No	1.00		
	Yes	1.03	0.67 - 1.57	0.899
Sex	Male	1.00		
	Female	1.37	1.09 - 1.72	0.007
Age (years)	6 - 18	1.00		
	< 6	0.68	0.37 - 1.28	0.233
	19 - 59	2.48	1.90 - 3.24	<0.0001
	≥ 60	4.36	2.93 - 6.50	<0.0001
Anthelmintic treatment, past year	No	1.00		
	Yes	0.83	0.66 - 1.04	0.102

Cough

Variable	Category	OR	95%CI	p-value
S. stercoralis infection	No	1.00		
	Yes	1.00	0.84 - 1.19	0.983
Hookworm infection	No	1.00		
	Yes	0.87	0.72 - 1.05	0.138
Other helminth infection	No	1.00		
	Yes	0.85	0.62 - 1.17	0.327
Pathogenic protozoa infection	No	1.00		
	Yes	0.73	0.55 - 0.96	0.024
Sex	Male	1.00		
	Female	0.99	0.84 - 1.17	0.931
Age (years)	6 - 18	1.00		
	< 6	1.06	0.77 - 1.48	0.708
	19 - 59	0.75	0.62 - 0.89	0.001
	≥ 60	0.85	0.60 - 1.19	0.345
Anthelmintic treatment, past year	No	1.00		
	Yes	1.00	0.84 - 1.18	0.974

Wheezing

Variable	Category	OR	95%CI	p-value
S. stercoralis infection	No	1.00		
	Yes	1.03	0.76 - 1.41	0.848
Hookworm infection	No	1.00		
	Yes	0.99	0.71 - 1.37	0.937
Other helminth infection	No	1.00		
	Yes	1.05	0.59 - 1.86	0.869
Pathogenic protozoa infection	No	1.00		
	Yes	0.61	0.30 - 1.21	0.156
Sex	Male	1.00		
	Female	1.28	0.95 - 1.73	0.111
Age (years)	6 - 18	1.00		
	< 6	0.77	0.32 - 1.86	0.566
	19 - 59	2.78	1.91 - 4.04	<0.0001
	≥ 60	4.17	2.46 - 7.09	<0.0001
Anthelmintic treatment, past year	No	1.00		
	Yes	0.92	0.68 - 1.23	0.559

Itching

Variable	Category	OR	95%CI	p-value
S. stercoralis infection	No	1.00		
	Yes	1.19	1.00 - 1.41	0.046
Hookworm infection	No	1.00		
	Yes	0.84	0.70 - 1.01	0.061
Other helminth infection	No	1.00		
	Yes	1.30	0.95 - 1.77	0.096
Pathogenic protozoa infection	No	1.00		
	Yes	0.89	0.68 - 1.18	0.416
Sex	Male	1.00		
	Female	0.91	0.78 - 1.06	0.234
Age (years)	6 - 18	1.00		
	< 6	0.75	0.55 - 1.03	0.075
	19 - 59	1.35	1.14 - 1.60	0.001
	≥ 60	1.62	1.17 - 2.26	0.004
Anthelmintic treatment, past year	No	1.00		
	Yes	1.22	1.03 - 1.43	0.018

Urticaria

Variable	Category	OR	95%CI	p-value
S. stercoralis infection	No	1.00		
	Yes	1.35	1.13 - 1.60	0.001
Hookworm infection	No	1.00		
	Yes	0.93	0.78 - 1.12	0.439
Other helminth infection	No	1.00		
	Yes	1.10	0.80 - 1.50	0.565
Pathogenic protozoa infection	No	1.00		
	Yes	0.94	0.71 - 1.25	0.681
Sex	Male	1.00		
	Female	0.93	0.79 - 1.09	0.38
Age (years)	6 - 18	1.00		
	< 6	0.57	0.41 - 0.80	0.001
	19 - 59	1.95	1.64 - 2.31	<0.0001
	≥ 60	1.64	1.18 - 2.28	0.003
Anthelmintic treatment, past year	No	1.00		
	Yes	1.50	1.27 - 1.77	<0.0001

Generalized rash

Variable	Category	OR	95%CI	p-value
S. stercoralis infection	No	1.00		
	Yes	1.20	0.97 - 1.48	0.089
Hookworm infection	No	1.00		
	Yes	0.74	0.59 - 0.94	0.014
Other helminth infection	No	1.00		
	Yes	1.12	0.76 - 1.65	0.562
Pathogenic protozoa infection	No	1.00		
	Yes	1.13	0.79 - 1.62	0.506
Sex	Male	1.00		
	Female	0.90	0.74 - 1.10	0.313
Age (years)	6 - 18	1.00		
	< 6	0.99	0.63 - 1.54	0.953
	19 - 59	2.28	1.81 - 2.87	<0.0001
	≥ 60	2.10	1.40 - 3.16	<0.0001
Anthelmintic treatment, past year	No	1.00		
	Yes	1.29	1.05 - 1.59	0.018

Fever

Variable	Category	OR	95%CI	p-value
S. stercoralis infection	No	1.00		
	Yes	0.98	0.83 - 1.17	0.851
Hookworm infection	No	1.00		
	Yes	0.94	0.78 - 1.13	0.507
Other helminth infection	No	1.00		
	Yes	0.91	0.66 - 1.24	0.536
Pathogenic protozoa infection	No	1.00		
	Yes	0.88	0.66 - 1.16	0.365
Sex	Male	1.00		
	Female	1.07	0.91 - 1.25	0.436
Age (years)	6 - 18	1.00		
	< 6	1.31	0.97 - 1.79	0.082
	19 - 59	0.76	0.64 - 0.90	0.002
	≥ 60	0.67	0.48 - 0.94	0.02
Anthelmintic treatment, past year	No	1.00		
	Yes	1.69	1.43 - 1.99	<0.0001

Tiredness

Variable	Category	OR	95%CI	p-value
S. stercoralis infection	No	1.00		
	Yes	1.04	0.84 - 1.28	0.750
Hookworm infection	No	1.00		
	Yes	0.79	0.63 - 0.99	0.041
Other helminth infection	No	1.00		
	Yes	1.54	1.06 - 2.24	0.023
Pathogenic protozoa infection	No	1.00		
	Yes	0.62	0.40 - 0.98	0.039
Sex	Male	1.00		
	Female	1.41	1.15 - 1.74	0.001
Age (years)	6 - 18	1.00		
	< 6	0.54	0.26 - 1.09	0.085
	19 - 59	4.71	3.63 - 6.12	<0.0001
	≥ 60	19.22	13.00 - 28.41	<0.0001
Anthelmintic treatment, past year	No	1.00		
	Yes	0.84	0.69 - 1.04	0.105

Muscle pain

Variable	Category	OR	95%CI	p-value
S. stercoralis infection	No	1.00		
	Yes	1.17	0.97 - 1.40	0.099
Hookworm infection	No	1.00		
	Yes	1.03	0.85 - 1.25	0.772
Other helminth infection	No	1.00		
	Yes	1.09	0.78 - 1.53	0.627
Pathogenic protozoa infection	No	1.00		
	Yes	1.05	0.76 - 1.45	0.760
Sex	Male	1.00		
	Female	0.79	0.66 - 0.94	0.009
Age (years)	6 - 18	1.00		
	< 6	0.22	0.12 - 0.41	<0.0001
	19 - 59	4.72	3.89 - 5.73	<0.0001
	≥ 60	4.86	3.45 - 6.84	<0.0001
Anthelmintic treatment, past year	No	1.00		
	Yes	1.56	1.30 - 1.86	<0.0001

Data were obtained from 2,744 participants in a cross-sectional survey carried out 2012 in eight villages of Preah Vihear province, Cambodia.

OR: odds ratio; CI: confidence interval.
