# Supplementary material for: Strongyloides stercoralis is associated with significant morbidity in rural Cambodia, including stunting in children
Source: PLoS Negl Trop Dis. 2017 Oct 23;11(10):e0005685. doi: 10.1371/journal.pntd.0005685 (PMC5695629; doi:10.1371/journal.pntd.0005685)
Supplement: S4 Table — Treatment: ivermectin 200 μg/kg BW. OR in bold were significant at 95% level. LRT p-values were obtained from conditional logistic regressions. Data were collected in 2012 in 2 villages of Preah Vihear Province, North Cambodia, from 208 S. stercoralis patients regardless of infection with any other diagnosed helminth or protozoa and met the cases definitions used in this work for all parasites. The total of 208 patients is constituted of 103 patients free of any infection other than S. stercoralis and 105 patients co-infected by any other parasite at any survey. OR: odds ratio; CI: confidence interval; LRT: likelihood ratio test. (PDF) [file pntd.0005685.s005.pdf]

S4 Table. Symptoms reported before and after ivermectin treatment by *S. stercoralis* infected patients including co-infection with other parasites (208 patients)

Symptom		OR	95% CI	LRT p-value
Loss of appetite / anorexia	before treatment	1.00		
	after treatment	**0.61**	0.40 - 0.95	0.027
	any other infection	1.69	0.70 - 4.07	
Abdominal pain	before treatment	1.00		
	after treatment	**0.07**	0.04 - 0.15	<0.0001
	any other infection	0.91	0.25 - 3.25	
Nausea	before treatment	1.00		
	after treatment	**0.27**	0.15 - 0.48	<0.0001
	any other infection	1.56	0.52 - 4.64	
Vomiting	before treatment	1.00		
	after treatment	**0.08**	0.03 - 0.27	<0.0001
	any other infection	2.66	0.23 - 30.71	
Diarrhoea	before treatment	1.00		
	after treatment	**0.42**	0.27 - 0.65	<0.0001
	any other infection	0.77	0.35 - 1.70	
Constipation	before treatment	1.00		
	after treatment	0.80	0.39 - 1.63	0.536
	any other infection	2.15	0.54 - 8.54	
Itching	before treatment	1.00		
	after treatment	1.04	0.70 - 1.55	0.841
	any other infection	1.55	0.72 - 3.30	
Urticaria	before treatment	1.00		<0.0001
	after treatment	**0.03**	0.01 - 0.08	
	any other infection	1.22	0.16 - 9.56	
Cough	before treatment	1.00		
	after treatment	**0.23**	0.14 - 0.38	<0.0001
	any other infection	1.26	0.51 - 3.11	
Wheezing	before treatment	1.00		
	after treatment	1.48	0.78 - 2.80	0.227
	any other infection	1.51	0.50 - 4.53	

Fever	before treatment	1.00		
	after treatment	1.16	0.76 - 1.78	0.486
	any other infection	1.57	0.70 - 3.49	
Tiredness	before treatment	1.00		
	after treatment	**0.49**	0.30 - 0.81	0.004
	any other infection	0.84	0.31 - 2.27	
Muscle pain	before treatment	1.00		
	after treatment	1.07	0.70 - 1.62	0.755
	any other infection	1.62	0.73 - 3.58	

Treatment: ivermectin 200 µg/kg BW.

OR in bold were significant at 95% level. LRT p-values were obtained from conditional logistic regressions.

Data were collected in 2012 in two villages of Preah Vihear Province, North Cambodia, from 208 *S. stercoralis* patients regardless of infection with any other diagnosed helminth or pathogenic protozoa and met the cases definitions used in this work for all parasites. The total of 208 patients is constituted of 103 patients free of any infection other than *S. stercoralis* and 105 patients co-infected by any other parasite at any survey.

OR: odds ratio; CI: confidence interval; LRT: likelihood ratio test.
